# From plant extract to molecular panacea: a commentary on Stone (1763) ‘An account of the success of the bark of the willow in the cure of the agues’

**DOI:** 10.1098/rstb.2014.0317

**Published:** 2015-04-19

**Authors:** John N. Wood

**Affiliations:** 1 Wolfson Institute for Biomedical Research, University College London, Gower St., London WC1E 6BT, UK; 2 Department of Molecular Medicine and Biopharmaceutical Sciences, College of Medicine, Seoul National University, Seoul, Korea

**Keywords:** pain, inflammation, malaria, fever, cardiovascular function, aspirin

## Abstract

The application of aspirin-like drugs in modern medicine is very broad, encompassing the treatment of inflammation, pain and a variety of cardiovascular conditions. Although anecdotal accounts of willow bark extract as an anti-inflammatory drug have occurred since written records began (for example by Hippocrates), the first convincing demonstration of a potent anti-pyretic effect of willow bark containing salicylates was made by the English cleric Edward Stone in the late eighteenth century. Here, we discuss the route to optimizing and understanding the mechanism of action of anti-inflammatory drugs that have their origins in Stone's seminal study, ‘An account of the success of the bark of the willow in the cure of agues’. This commentary was written to celebrate the 350th anniversary of the journal *Philosophical Transactions of the Royal Society*.

## 1. Introduction

In 1763, Edward Stone wrote to the Earl of Macclesfield, then President of the Royal Society, with ‘An account of the success of the bark of the willow in the cure of agues’ [1]. This landmark paper is the basis for the discovery of the actions and chemical structure of aspirin, and the development of non-steroidal anti-inflammatory drugs, known as NSAIDs, that are so useful in treating inflammatory pain and fevers. However, at the time of publication, the most problematic ‘ague’ (fever) was probably that caused by malaria. Stone's observations eventually led to the discovery that aspirin-like drugs act to suppress the product of lipid mediators derived from arachidonic acid that play a key role in cardiovascular function as well as the regulation of pain thresholds during inflammation.

**Figure 1.**
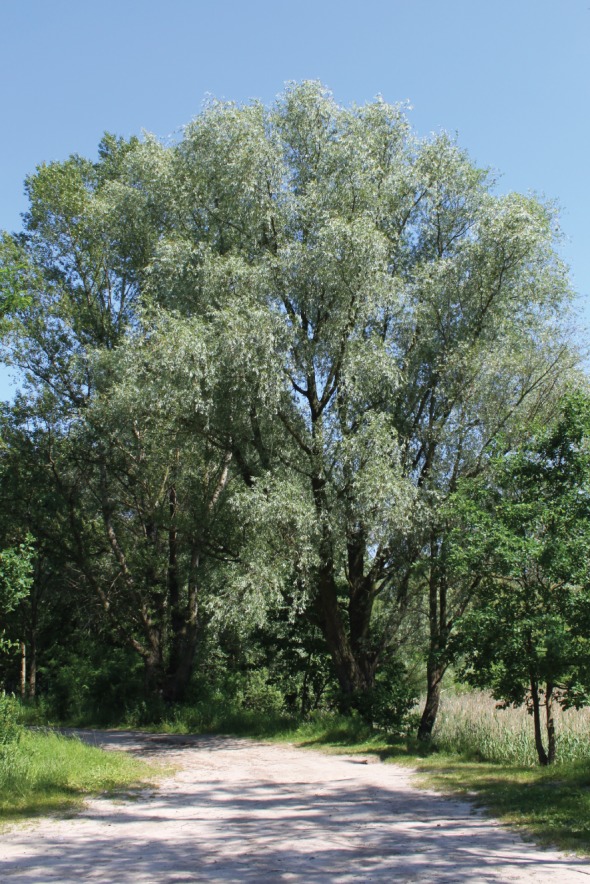
The white willow tree (*Salix alba*) was the source of salicylates used by Edward Stone. However, many other plant species also produce these compounds. (Image: ‘*Salix alba* Marki 2’ by Crusier—Own work. Licenced under Creative Commons Attribution-Share Alike 3.0 via Wikimedia Commons).

Among the many plant products that have clinical utility, salicylates and aspirin are probably the most broadly used (estimates suggest about 40 000 tons a year) and they are recognized as effective agents for the treatment of a broad range of painful maladies [2]. Historical records demonstrate that bark extracts were used medicinally in the ancient world, in South America, Egypt, Classical Greece and China. Remarkably, Hippocrates in the fourth century BC was aware of the utility of willow bark in the treatment of inflammatory pain [3] (figure 1). Nevertheless, the relatively recent study published by Edward Stone in *Philosophical Transactions of the Royal Society* in 1763 has a special significance in terms of its rigorous scientific approach, its description of an early clinical trial and its impressive demonstration of the utility of willow bark in the treatment of a range of fevers or agues.

Edward Stone (misnamed Edmund on his willow bark paper) lived in Chipping Norton, an Oxfordshire village, where he held a position as chaplain to Sir Jonathan Cope at nearby Bruern Abbey [4]. He contributed to the eighteenth century passion for new scientific approaches to understanding the natural world in many ways, including studies of the movement across the sun of the planet Venus in 1761. Edmund Halley had suggested that studies of the transit of Venus could give an accurate measurement of the size of the solar system and Stone published a related article, ‘The whole doctrine of parallaxes explained and illustrated by an arithmetical and geometrical construction of the transit of Venus over the Sun, June 6th, 1761’.

During the eighteenth century, malaria was endemic in England and Western Europe, probably due to transmission by the mosquito *Anopheles atroparvus* living in estuarine marsh lands [5]. However, an extract of the bark of the South American cinchona tree had been shown to be an effective therapeutic agent for malaria treatment. Quechua Indians who had deduced that tree extracts were useful for suppressing shivering as well as treating malaria told Jesuit missionaries, who introduced Peruvian bark or ‘Jesuits bark’ to Europe in 1632 [6]. The active ingredient was the alkaloid quinine, an anti-malarial compound that acts to kill malarial parasites. Quinine probably causes the accumulation of haem in malaria parasites leading to their death, but the mechanism of action remains incompletely understood.

Religious and political intrigue coloured the exploitation of this important discovery that Edward Stone would certainly have been aware of, as the London Pharmacopoeia of 1677 refers to Peruvian bark. Interestingly, Oliver Cromwell probably died of malaria and is supposed to have refused treatment with Peruvian bark because of its Jesuitical origins [7]. Remarkably, both the English and French royal families also suffered from malaria. The association of the Peruvian bark with the Jesuits (with a presumed interest in poisoning Protestants) meant that King Charles II treated his malarial fever discretely. His physician, Robert Talbor, was knighted and became a member of the Royal College of Physicians (but not the Royal Society!) as the result of an entirely successful treatment of the King's malaria with a secret potion that Talbor claimed to be his invention, but which actually contained bark-derived quinine. Showing no religious discrimination in his activities, in 1679, Talbor successfully also treated the Dauphin son of Louis XIV and was rewarded with 2000 gold crowns and a lifetime pension on condition that his secret potion should not be revealed until after his death. When Talbor died aged only 39, the secret medicine was found by the French King to comprise 7 g of rose leaves, 2 ounces of lemon juice and cinchona bark, extracted with wine to purify the active alkaloids quinine and quinidine. In 1681, the Marquise de Sevigne, a noted literary figure revered by the French establishment, wrote that it would soon become the fashion to take Jesuit's bark after every meal, like tea or chocolate [8].

Further evidence that the English knew about Jesuit bark and its useful properties comes from the weekly journal *Mercurius Politicus*, which in 1658 announced: ‘The excellent powder known by the name of ‘Jesuit's powder’ may be obtained from several London chemists’. The Spanish had a monopoly on Jesuit Bark but a large amount of material was captured by the English gentleman pirate Basil Ringrose, who was eventually killed by the Spanish in 1686 [9]. From the late seventeenth century onwards, the import of Peruvian bark into England exploded, with the import of enough material to treat hundreds of thousands of people [10,11]. Thus, the use of a bitter tree bark extract as a useful medicine was well established in England and Europe many years before Stone's paper which encompasses malaria (binary ague) as a malady that can be treated.

Stone's paper has caught the public imagination, not just because of the clear utility of its findings but also because of the fascinating narrative style used by the author. Many years’ study contributed to the findings and the claims made have been vindicated in the following centuries. Stone states that his discovery, among many made at the time, deserves the attention of the public. He relates his discovery around 1656 that the bark of an English tree, the common white willow, had the same bitter taste as the Peruvian bark, which raised his suspicions that the same useful properties might be found in willow bark. He also noted that the tree delights in moist soil and cites the general maxim that many natural maladies have remedies that exist not far from their causes:

… that many natural maladies carry their cures along with them, or that their remedies lie not far from their causes, was so very apposite to this particular case that I could not help applying it … [2; p. 195]

By this he may well have meant the cure of malaria, as much as the treatment of rheumatic diseases. Stone first did the equivalent of a Medline search to see if anyone else had discovered a use for willow bark, but could only find references to the tree's name. He then gathered a pound of willow bark and left it next to a baker's oven for three months, before pulverizing and sifting the bark into a fine powder. In the best traditions of pioneering medicine [12,13], Stone then tried the powder on his own fever, first eating 20 grains, and when no ill effects were apparent moving up to two scruples. A word on doses is appropriate here. The use of grains does indeed refer to the weight of a single grain of barley, considered equivalent to 1^1^/_3_ grains of wheat in the ancient English system of weights and now defined faintly absurdly as 64.79891 mg, so that an avoirdupois pound comprises 7000 grains. A scruple is 20 grains, equivalent to about 1.3 g, and a much heavier dose. Stone found that his ague was abolished on treatment every 4 h, although whether he was suffering from malaria or another rheumatic disorder is not clear. He recommended a dose of a dram—equal to about 3.6 g of willow bark extract (the purity of which was unknown but is in the region of 2% salicylates) that were tested on several other people. Over the next 5 years, Stone tested the powder on around 50 people who benefited from a cure, although a few with quartan agues (fever every 4 days) only partially recovered from the fever. Stone next added a fifth part of the Peruvian bark and found that the ague was completely routed. This suggests that the fever was the result of malaria in these cases.

Stone points out that his subjects were not on other medication and had not been prepared by bleeding or vomiting before taking the bark—usually with water, tea or small beer. Importantly, he also notes that unlike Peruvian bark, his willow bark extracts had no ill effects.

Why should plants produce so many useful medicinal compounds for the treatment of human disease? One thinks of opium, not to mention the various psychoactive materials that have enriched South American indigenous culture. A selective advantage for plants that produce various pain-reducing compounds has been proposed, including the idea that opioids are found on parts of plants that are easy to access by herbivores, and hence may facilitate their dispersal by animal means. Whether salicylates contribute to plant dispersal through animal activity is uncertain but a highly significant role for salicylates in plant physiology has also been established over the past 50 years. Plants seem to have exploited salicylic acid in inducible defence systems. Salicylates are probably derived from the degradation of cinnamic acids, which are part of the shikimic acid pathway. Salicylates have been invoked as resistance mediators for pathogens and phloem-feeding insects that act through the induction of a number of proteins involved in host protection and which are capable of establishing an acquired resistance to various pathogens. Shulaev *et al*. [14] showed that methyl salicylate, commonly known as oil of winter-green, is produced as a volatile liquid by tobacco plants inoculated with tobacco mosaic virus. The methyl salicylate is synthesized from salicylic acid and can be dispersed through the air. Methyl salicylate then acts by being converted back to salicyclic acid in other nearby plants in which it carries out its protective functions. Methyl salicylate, found in many plants, may therefore be an airborne signal which activates disease resistance through the expression of defence-related genes in neighbouring plant tissue [15]. It is fascinating to see the diversity of functions that aspirin-related compounds control in plants and animals and to consider the evolutionary mechanisms that may underpin the high levels of expression of salicylates in many plants.

Interestingly, willow bark extract is still used as an over-the-counter medicine for inflammatory disorders, although the transition from folk medicine plant extract to defined chemical structure (and lucrative medicine) occurred more than 150 years ago in France and Germany. The active ingredient of willow bark, salicylic acid, was first isolated by Johann Andreas Buchner in 1827 and 2 years later Henri Leroux managed to obtain about 30 g of purified salicin from 1.5 kg of bark [16]. In 1853, the Frenchman Charles Gerhardt who worked as a chemist in Germany and later in Paris at the Jardin des Plantes synthesized a form of acetyl salicylic acid as part of his studies on anhydrides [17]. His career was terminated by an early death at age 39, ascribed to poisoning resulting from his activities in synthetic chemistry.

Related work by von Gilm in 1859 led to a pure version of acetyl salicylic acid, the structure of which was accurately described in 1869 by Schröder *et al*. [18] who repeated the studies of Gerhardt and von Gilm. Some years then elapsed before the dramatic developments at Bayer that made aspirin a household name and provided the remarkably efficacious formulation that is still in daily use today. In 1897, Bayer chemists produced a stable acetylated salicylate from salicylic acid derived from the meadowsweet plant rather than willow bark. The compound named aspirin was a contraction of the two words acetyl and *Spirsäure—*or salicylic acid. There is controversy about who was principally responsible for the production of aspirin. The accepted story is that Felix Hoffman was anxious to treat his father's arthritis without using large doses of sodium salicylate, which although effective as an anti-arthritis drug was known to cause stomach pain and irritation. On 10 August 1897, Hoffmann described the synthesis of acetylsalicylic acid (ASA) in a pure form suitable for medical use. However, it now seems that aspirin had been produced and tested at Bayer earlier by the distinguished chemist Arthur Eichengrun, who directed Hoffman's work, and who was made Head of Pharmacology research at Bayer shortly after this discovery. Eichengrun left Bayer to start his own very successful chemical company, but suffered like other Jews in the Nazi period, and was lucky to survive the Second World War as a prisoner. He claimed that his contribution to the development of aspirin had been expunged from the archives during the Nazi period, and this view is supported by Walter Sneader who analysed Eichengrun's contribution to the development of aspirin in detail [19]. However, Bayer continued to highlight the role of Hoffman in aspirin's success.

Aspirin was marketed by Bayer and enjoyed enormous success in the early nineteenth century for the treatment of pain, rheumatic fever and inflammatory diseases such as rheumatoid arthritis, although war reparations in 1919 meant that the term Aspirin became a generic term used for material supplied by many other pharmaceutical companies after World War 1.

Having discovered the active ingredient of willow bark that has such useful medicinal qualities and established the chemistry necessary to produce large amounts of this material, the mechanism of action of aspirin and related drugs remained uncertain until 1971. The activities of a research team at the Wellcome Foundation in Beckenham led by John Vane provided a mechanistic explanation for the utility of not only aspirin, but also other members of the drug family defined as NSAIDs [20]. Vane and colleagues showed that aspirin and related drugs inhibit the action of key enzymes (cyclooxygenases, COXs) involved in arachidonic acid metabolism. COX, formally named prostaglandin-endoperoxide synthase, is an enzyme that catalyses the first step in the formation of a diverse family of eicosanoids, which are very significant signalling molecules derived from the 20-carbon fatty acid arachidonic acid.

**Figure 2.**
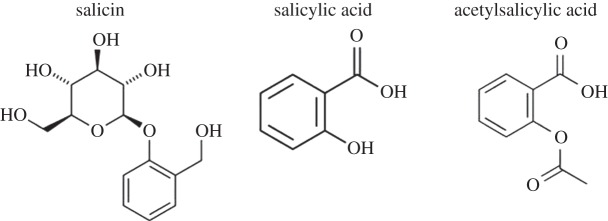
Salicin is the precursor of salicylic acid, which in its more palatable form as ASA is the pain-killing ingredient in aspirin.

**Figure 3.**
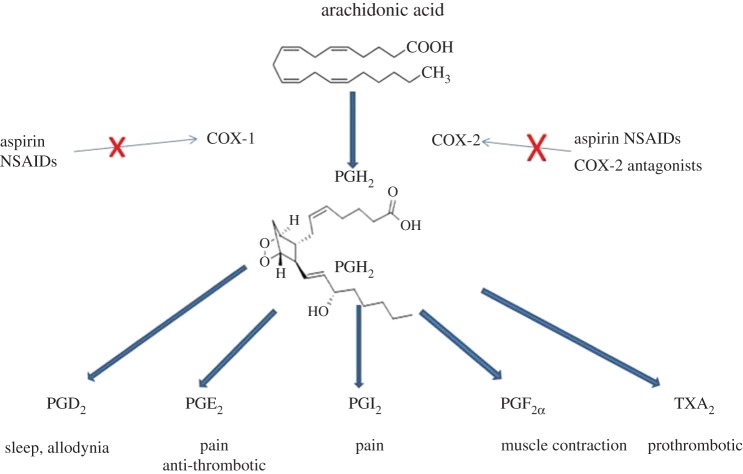
The COX enzymes generate PGH_2_, the precursor of a number of arachidonic acid metabolites that have roles in regulating both cardiovascular and pain systems. Aspirin and NSAIDS block both COX-1 and COX-2. TXA_2_, thromboxane A_2_.

**Figure 4.**
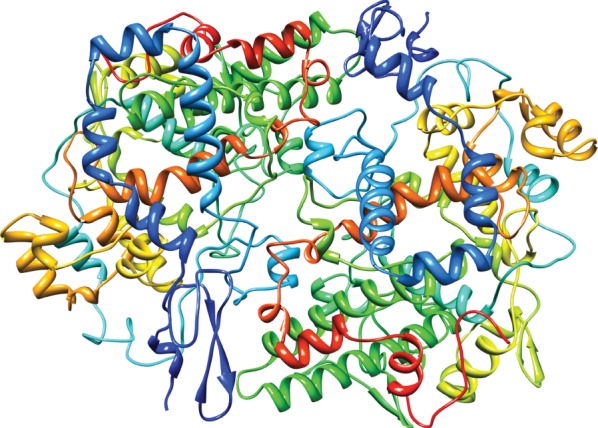
Crystallographic structure of prostaglandin H_2_ synthase-1 complex with flurbiprofen [21]. (Image: Prostaglandin H_2_ Synthase-1 Complex PDB 1CQE. doi:10.2210/pdb1cqe/pdb).

Two closely related forms of the enzyme have been cloned and characterized; these enzymes are usually referred to as COX-1 and COX-2. The tissue distribution and level of expression of these two enzymes is quite distinct; the COX-1 enzyme is primarily constitutively expressed, while COX-2 activity is induced in a variety of inflamed states after tissue injury. COX enzymes catalyse the peroxidation of arachidonic acid to a labile intermediate prostaglandin (PG)G_2_, which is then reduced to PGH_2_ by the enzyme's peroxidase activity. PGH_2_ is then a substrate for a variety of enzymes that produce different prostaglandins that, acting through G-protein-coupled receptors (GPCRs), modulate many aspects of the inflammatory process (figures 2–4). Aspirin acetylates and irreversibly blocks the activity of COX-1, thus shutting down thromboxane production from platelets, which are unable to synthesize fresh enzyme for the lifetime of the platelet. Aspirin also inhibits COX-2 activity, although the arachidonic acid derivative 15-HETE can still be produced from the aspirin–COX-2 complex.

Interestingly, some eicosanoid products produced downstream of COX enzymes have opposing effects on cardiovascular functional activity. For example, the short-lived thromboxane A_2_ produced by thromboxane A synthase is released from activated platelets and causes platelet aggregation as well as vasoconstriction through GPCR-mediated actions. Thus, clotting is potentiated by thromboxane A_2_. By contrast, prostacyclin (PGI_2_), another short-lived eicosanoid product of PGH_2_ produced by prostacyclin synthase in endothelial cells, was shown by Moncada [22] to be a potent vasodilator that also inhibits platelet aggregation. The useful effects of low-dose aspirin in diminishing heart attacks have been ascribed to its actions in lowering the levels of pro-thrombotic thromboxane A_2_ [23].

Aspirin use has been associated with stomach bleeding and gastrointestinal pain, and COX-1 is expressed within the gastric mucosa. This knowledge led to attempts to selectively block COX-2 in an attempt to generate drugs that had anti-inflammatory and pain-killing actions without the associated gastrointestinal side effects of aspirin. Structural differences in the two COX enzymes enabled selective COX-2 antagonists to be generated. At position 523 in human COX-2 enzymes there is a large cleft, which allowed drugs to be developed that access and selectively block COX-2 but not COX-1 [24]. However, the development of COX-2 selective antagonists led to major cardiovascular problems including increased heart attacks and stroke. The reason for this is that endothelial cells seem to express COX-2 and release prostacyclin, the anti-thrombotic factor, while platelets that produce pro-thrombotic thromboxane A_2_ express mainly COX-1. Selective inhibition of COX-2 may lead to an imbalance of these two key mediators, so that thromboxane may trigger deleterious consequences. The potential dangers of selective COX-2 antagonists were thus known to result in cardiovascular problems that outweighed the benefits of better gastrointestinal tolerance. These observations led to the withdrawal of most COX-2 inhibitors as anti-inflammatory agents [25,26].

What of the future? Aspirin has been found to acetylate a range of molecules, not just COXs, and new applications in the cancer field are now under investigation [27]. Some evidence supports an anti-cancer effect of aspirin in some colon and other gastrointestinal tract tumours. A number of workers have suggested that aspirin may interact and acetylate not only enzymes but molecules such as RNA, and metabolites such as coenzyme A, leading to a change in their function [28]. Thus, aspirin research is still an active topic with considerable significance for human health.

Stone concluded that the motives for publishing such a minute account of his discovery resulted from (to paraphrase) the wonderful efficacy of the extract in treating fevers that are fully supported by his manifold experience of it, and hopes that the world may reap the benefits of his discovery. His brief paper has indeed had the effect that Stone desired.

## Author profile

**John N. Wood** is Professor of Molecular Neurobiology at University College London. He spent 12 years in industry, first at the Wellcome Foundation where the mechanism of action of aspirin-like drugs was unravelled, and later at Novartis, before founding the Molecular Nociception Group at UCL in 1995. There he has concentrated on peripheral sensory neurons as targets for pain medication, exploiting both mouse and human genetics to find new drug targets. His group has focused on voltage-gated sodium channels in peripheral pain pathways and has been addressing some of the pain problems mentioned in the accompanying article. He won the Grand Prix Scientifique of the Institut de France in 2009 and became a Fellow of the Royal Society in the same year.
